# Knowledge and Attitudes Regarding Breast Cancer Screening and Mammograms Among Women Aged 40 Years and Older in the United Arab Emirates

**DOI:** 10.7759/cureus.59766

**Published:** 2024-05-06

**Authors:** Tazeen Afroze, Aashka Iyer, Hana Faisal, Hiba Manaf, Radha Bahul

**Affiliations:** 1 Family Medicine Department, Nad Al Hamar Health Center, Dubai, ARE; 2 Community Medicine Department, Gulf Medical University, Ajman, ARE

**Keywords:** knowledge attitude, breast cancer screening barriers, : “breast cancer”, mammogram screening, awareness knowledge and practice

## Abstract

Objectives: To assess the knowledge and attitude regarding breast cancer screening and mammograms among 40 years and older females in the United Arab Emirates.

Methods: A cross-sectional questionnaire-based study was conducted on women faculty, staff, and female patients attending our hospital. The inclusion criteria were women ≥ 40 years old who agreed to participate. The exclusion criteria were women < 40 and those ≥ 40 years who refused to participate. A signed informed consent was taken. A p-value of < 0.5 was considered significant.

Results: Among the 460 women enrolled, 420 completed the survey (response rate 91%). The mean age was 48.4 ± 8.2 years. A total of 63.4% of the participants were < 50 years of age. A total of 53.3% were never screened before. About 98% believed that screening is beneficial in early detection. Social media (52.2%) and health professionals (46%) played a vital role in creating awareness. The majority of women were aware of self-breast examinations (73.3%), followed by mammography (68.6%). About 84% and 68.3%, of the participants had incorrect knowledge of the timing and frequency of mammograms, respectively. Only 16.3% of the participants were recommended by their physician, while the rest (83.7%) performed screening based on their awareness. No significant association was found between nutritional status (p=0.252), age at first pregnancy (p=0.409), or having children (p= 0.377) with mammogram uptake. There was a significant association between the perceived benefit of screening and mammogram uptake (p=0.033). There was a positive association between radiation therapy to the chest area and mammogram uptake (p<0.024). A statistically significant association was found between the correct timing of mammograms with family history of cancer (p = 0.037) and previous exposure to radiation therapy to the chest (p = 0.002).

Conclusion: There is a need to increase knowledge and awareness regarding breast cancer screening and mammograms among women in UAE. Specifically, breast self-examination should be encouraged and recommended.

## Introduction

Cancer is the leading cause of morbidity as well as mortality across the globe [1]. It’s the most important cause of non-communicable, disease-related mortality worldwide and ranked second highest in the United Arab Emirates (UAE) [2]. UAE has a cancer incidence rate of 38.8% and a resulting mortality rate of 11.8% [3]. Among both genders, the most commonly diagnosed cancer and the leading cause of cancer-related death is lung cancer followed by breast cancer [1]. Breast cancer (BC) accounts for the highest incidence rate of cancer in women worldwide [4]. It is the most frequent cancer in females among UAE citizens [5]. With rapid population growth and aging, cancer-related deaths are on the rise worldwide as well as in the UAE [1]. Females in the UAE have a tendency to develop BC at least a decade earlier than their counterparts in Western countries [5].

Screening is the most effective method to reduce mortality and morbidity from breast cancer [5-8]. It is the key element widely recommended by professional guidelines and supported by organized screening programs in several countries around the globe [8]. Variations in adherence to breast cancer screening percentages have been reported in different ethnic countries depending on various factors [9]. Screening methods like breast self-examination (BSE), clinical breast examination (CBE), and mammography have been defined as methods facilitating the early detection and improvement of women’s health [5-8]. Lack of awareness about screening methods and potential barriers that individuals face have been major factors contributing to the delayed diagnosis of breast cancer, reducing survival rates in diagnosed patients [5]. The American Cancer Society advises women aged 40 and above to get screened at least once annually [10].

Various studies have shown that there is a lack of awareness among women about breast cancer screening methods. There are gaps in the knowledge of factors deterring women in the UAE from receiving breast examinations and mammograms, and due to the limited research conducted on screening uptake in the UAE, it is important to compile information about this phenomenon. Keeping this in mind, we conducted this research in our hospital to collect information about knowledge, awareness, and practices for breast cancer screening and mammograms as a screening method, and to evaluate barriers or limitations causing nonadherence, which should facilitate the development of effective interventions. 

## Materials and methods

A cross-sectional study was conducted on all women faculty, staff, and female patients attending our hospital. The duration of the study was 2 months. The inclusion criteria was women ≥ 40 years old who agreed to participate. The exclusion criteria were women below the age of 40 and those ≥ 40 years who refused to participate. A signed informed consent was taken. This study was approved by IRB (IRB/COM/STD/05/Nov-2021) and followed the Declaration of Helsinki.

The sample size was calculated assuming the prevalence of screening for breast cancer and awareness about mammograms in UAE to be 85% [11]. Considering a population size of 1 million, a 95% confidence level, and a design effect of 2 %, a total of 385 participants are required. Considering the 10% data loss, the sample was increased to 420 participants.

The study instrument was a self-administered questionnaire (Appendix material-1). A questionnaire was developed to assess knowledge and attitudes regarding breast cancer screening uptake among women in UAE [12-13]. The questionnaire was validated by three experts in the field. A pilot study was done among five participants. The final questionnaire was delivered to eligible women. The research objectives were explained to them. Women who had accepted to participate and signed informed consent were handed the final questionnaire to be filled.

Data was entered into Excel and the furthest statistical analysis was done using SPSS version 27. Data was presented in the form of tables and figures. The chi-square test was used to find the association between the variables, and logistic regression analysis was used to find out predictors of screening uptake. A p-value of less than 0.5 was considered as significant.

## Results

**Figure 1:**
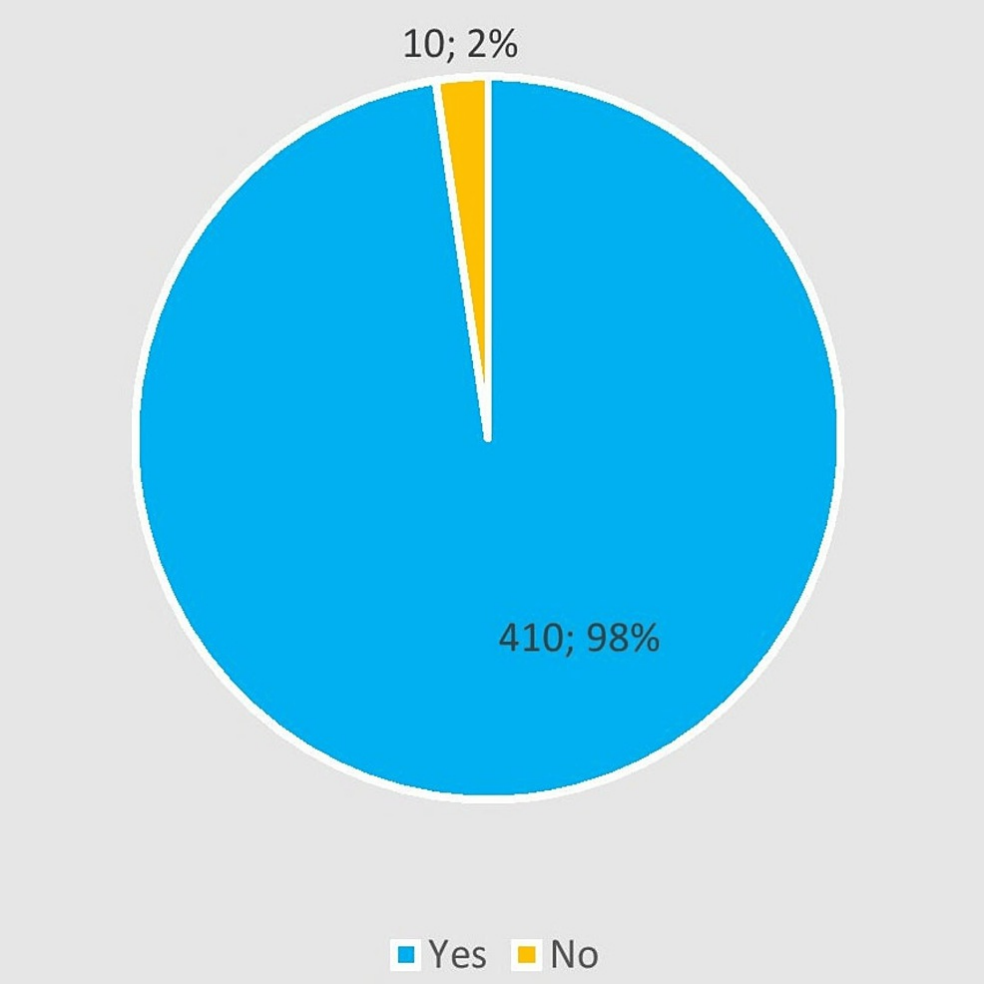
Perceived benefit of screening Distribution of participants by perceived benefit of screening (N= 420)

Among the 460 enrolled participants, 420 completed the survey (response rate 91%). The mean age was 48.4 ± 8.2 years ranging from 40 to 81 years. A total of 63.4% of the participants were under the age of 50 years. Among all participants, only 196 (46.66%) had a previous breast cancer screening, while the remaining 224 (53.3%) were never screened before. Most of the participants (98%) believed that screening for breast cancer is beneficial in early detection (Figure 1).

Social media (52.2%) and health professionals (46%) played a vital role in creating awareness about breast cancer screening followed by advertisements (32.2%). Family (24.7%) and friends (15.7%) had the least participation in creating awareness (Table 1).

**Table 1:** Source of awareness reported by the participants

	Number	percentage
Social media	215	52.2
Health professional	190	46
Advertisement	133	32.2
Family	102	24.7
Friend	65	15.7

**Figure 2:**
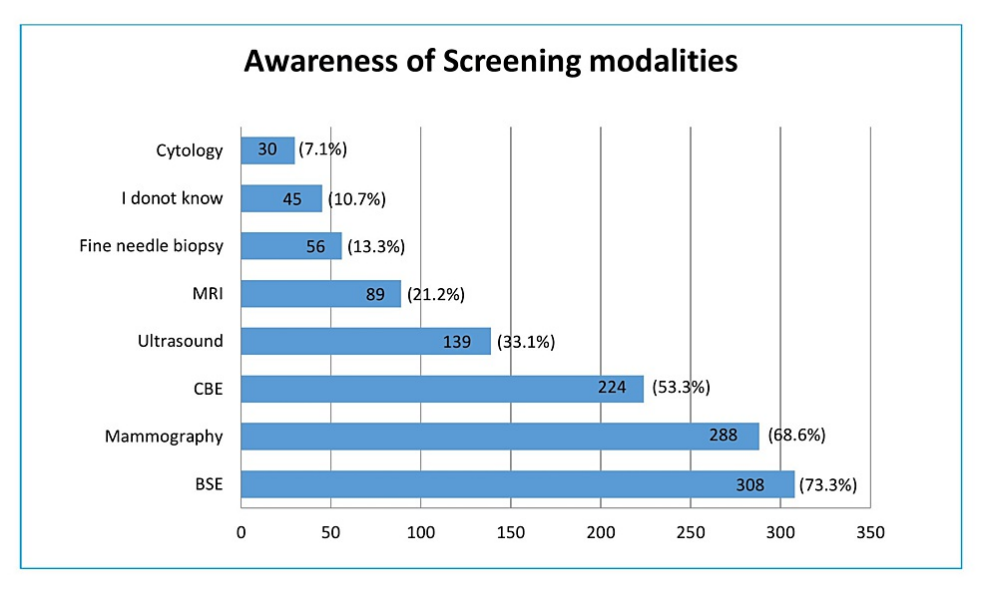
Awareness of screening modalities reported by participants

The majority of women were aware of self-breast examinations (73.3%) and mammography (68.6%), while only 10.7% of the participants had no idea about screening modalities (Figure 2).

The majority (84%) of the participants had incorrect knowledge of the timing of conducting a mammogram, believing it to be later than it should be. More than half (68.3%) of the participants had correct knowledge about how often mammograms should be done. About 67.1% of the participants had correct knowledge about how often CBE should be conducted. The majority of the participants (87.2%) stated the incorrect stage of the menstrual cycle at which BSE must be conducted (Table 2).

**Table 2:** Awareness regarding timing and frequency of screening

Variable	Response	Number	percentage
Age of starting mammogram	Correct knowledge	67	16
	Incorrect knowledge	352	84
Frequency of mammogram	Correct knowledge	286	68.3
	Incorrect knowledge	133	31.7
Frequency of CBE	Correct knowledge	282	67.1
	Incorrect knowledge	138	32.9
BCE done in which part of menstrual cycle	Correct knowledge	37	12.8
	Incorrect knowledge	251	87.2

**Figure 3:**
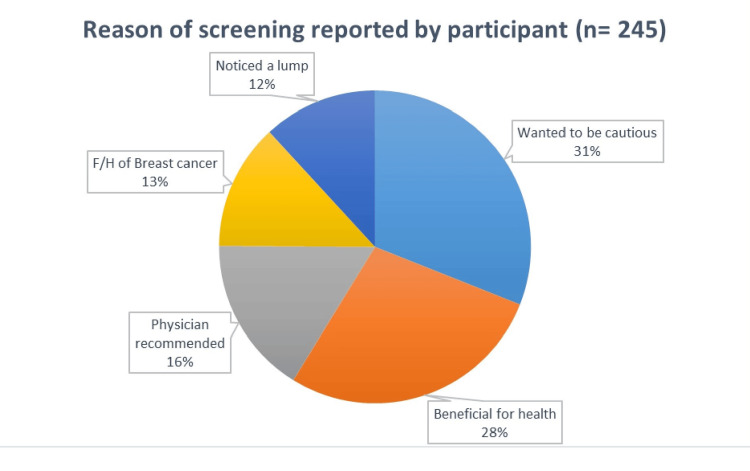
Reason of screening reported by participants

Only 16.3% of the participants were recommended by their physician for screening; the rest of the participants 83.7% did screening based on their awareness (Figure 3).

The association between mammogram uptake with variables is shown in Table 3. No significant association was found between nutritional status and uptake of mammograms (p = 0.252). The majority of the participants who had undergone mammogram screening were overweight or obese. There was no significant association between having children and the uptake of mammograms (p= 0.377). The majority of the participants (68.7%) in our study who had children did not participate in mammogram screening for breast cancer. Age at first pregnancy and mammogram uptake were not significantly associated (p=0.409). Most of the women who participated in mammogram screening were first pregnant when they were in the age group of < 25 years. There was a significant association between the perceived benefit of screening and mammogram uptake (p=0.033). All the participants in our study who had undergone mammogram screening perceived screening for breast cancer to be beneficial for health. There is a positive association between radiation therapy to the chest area and mammogram uptake (P<0.024). Among the participants who had radiation therapy to the chest area, only 41% screened for breast cancer using mammograms.

**Table 3:** Association between mammogram uptake and variables

Variable	Groups	Uptake of mammogram screening				P value
		Yes		No		
		Number	%	Number	%	
BMI	Normal	31	27	84	73	0.252
	Obese/overweight	98	32.8	201	67.2	
Children	Yes	120	31.3	263	68.7	0.377
	No	9	24.3	28	75.7	
Age of first pregnancy	< 25 yr	72	33.5	143	66.5	0.409
	>25 yr	49	29.5	117	70.5	
Perceived benefit of screening	Yes	129	31.5	281	68.5	0.033
	No	0	0	10	100	
Radiation therapy to chest area	Yes	33	41.3	47	58.8	0.024
	No	96	28.3	243	71.7	

A statistically significant association was found between the correct timing of mammograms and the participants who had a family history of breast/pancreatic/ovarian cancer (p = 0.037). There was a significant association between the correct timing of mammograms and previous exposure to radiation therapy to the chest (p = 0.002). This measures the relationship between radiation therapy to the chest and uptake of screening at the correct age (Table 4, 5).

**Table 4:** Association of timing of mammogram with a family history of cancer

Variable	Groups	Family history of cancer				P value
		Yes		No		
		Number	%	Number	%	
Screened for Mammogram	Correct Timing	18	26.9	49	73.1	0.037
	Delayed	57	16.2	295	83.8	

**Table 5:** Association of timing of mammogram with history of radiotherapy to the chest

Variable	Groups	History of radiotherapy to the chest				P value
		Yes		No		
		Number	%	Number	%	
Screened for Mammogram	Correct Timing	22	32.8	45	67.2	0.002
	Delayed	58	16.5	293	83.5	

## Discussion

About less than half of the participants had undergone screening at least once in their lives by any modality. A study conducted in the year 2020 among Saudi women revealed that two-thirds of the participants in the study had never undergone screening for breast cancer [14]. Although awareness for breast cancer screening in UAE is better in our geographic region, it is one of the top three leading causes of death in UAE [1-2,5]. The best-documented technique for early diagnosis and treatment of breast cancer is a combination of regular breast examinations and mammograms [15]. Screening is an effective tool that allows for the detection and treatment of breast cancer at an early stage. Women who undergo routine mammograms have a 10% to 25% less chance of dying of breast cancer than women who do not have mammograms [5]. The majority of the participants in our study held the opinion that screening was an efficient tool for diagnosing cancers at an earlier stage. Regardless of uptake, they believed that undergoing routine screenings according to the recommended timings would be beneficial to their health. The findings of our study are similar to the results of a study conducted in Jordan which reported women believe that screening is a beneficial tool that can help in detecting breast cancer at an early stage [16]. We recommend increasing awareness campaigns in UAE.

The majority of the participants derived their information from social media while the second most highly stated source of awareness for breast cancer screening was doctors and health care providers. Although family and friends also played a role in awareness of screening, they lag behind. The results of the study are in agreement with a study conducted in Saudi Arabia which showed that the most common source of knowledge is social media followed by print media and physicians [17]. Although social media, physicians, and advertisements are doing their part, family and friends should also take the opportunity and spread awareness.

The results of our study indicated that only 16% of women had correct knowledge about how often mammograms should be performed for their age. Similar to the results of our study, a study conducted in Saudi Arabia revealed that only 25% of the participants were aware of the recommended age of screening [17]. The results of our study indicated that 68.3% of participants had correct knowledge about the frequency of mammogram screening. The awareness of our participants for correct knowledge regarding the frequency of mammogram screening is higher than the results of an Iranian study, which reported that 61.2% of females in the study were not aware of how often mammography should be done. The awareness regarding CBE and how often it should be performed is higher in our study (67.1%) compared to a study conducted in Saudi Arabia, which revealed that only 53.1% of women were aware of clinical breast examination as a screening modality and only 26.6% knew that this procedure must be done yearly [17]. A considerable number of women were not aware of the recommended time for performing BSE. The results of our study were not consistent with the findings of a Jordanian study, which reported that 50% of the participants practiced BSE after menstruation, 7.1% performed it during menstruation, and 8.7% performed it before menstruation [16]. We need to create awareness of the correct timing of BSE.

When asked about the reason for approaching screening, about one-third said they wanted to be cautious and equally felt that it would be beneficial for their health. Physician recommendation played for approaching screening a role in less than one-fifth of them. Few participants were encouraged due to a family history of breast cancer or they noticed a lump. The findings of our study are higher when compared to a study conducted to assess the factors affecting screening uptake among Asian women, as they were asked to do so by a healthcare professional [15]. The results of our study are higher when compared to an Iranian study which reported that 33.2% of female participants underwent screening because they wanted to be cautious and 21% of females underwent screening because they had a family history of breast cancer. However, the results for the participation of Iranian women in screening because they noticed a lump (14.8%) is consistent with the results of our study [18].

Regarding awareness of various screening modalities, more than half of the participants were aware of BSE, followed by mammography and CBE. A study conducted to assess the awareness and attitudes toward breast cancer screening among Japanese women above the age of 45 revealed that 86% of women believed that mammograms, CBE, and BSE are effective diagnostic tools [19]. The awareness regarding CBE was higher in our study compared to Saudi Arabia [17]. Less than half of them knew about other modalities like ultrasound, MRI, fine needle aspiration biopsy, and cytology. About ten percent of participants have no idea of screening modalities. These are the people for whom an awareness drive is required and recommended.

There was no significant association between the BMI of the participants and the uptake of mammograms. Our study revealed that more than two-thirds of the participants were categorized as overweight or obese, and a majority had not undergone screening for breast cancer. On the contrary, a study conducted in Italy found a correlation that obese women had a lower likelihood of having a mammogram than normal-weight women [20]. The current study found no significant association between a woman’s having children and mammography screening uptake. However, a study conducted in Saudi Arabia regarding the patterns of mammography use found that women having < 2 children were less likely to have a mammogram [21]. There is no significant association between age at first pregnancy and uptake of mammograms. Most of the women who participated in mammogram screening were first pregnant when they were in the age group of < 25 years. There was a significant association between the perceived benefit of screening and the uptake of mammograms. All the participants in our study who had undergone mammogram screening perceived screening for breast cancer to be beneficial for health. About two-thirds of those who did not screen using mammograms also believe that there is a benefit of mammograms. There is a positive association between radiation therapy to the chest area and mammogram screening uptake. Among the participants who had radiation therapy to the chest area, only 41% screened for breast cancer using mammograms.

The current study revealed that a family history of breast/pancreatic/ovarian cancer is significantly associated with a higher likelihood that an individual would undergo a mammogram at the correct time. There is a higher chance of lower uptake of mammograms in people without a family history. These results are reflected in a study conducted in Egypt in which women with a family history of breast cancer were more likely to perceive fewer barriers and overcome any perceived barriers, than those who had no family history [22]. Another survey conducted in the United States corroborated our findings, indicating that cancer risk perception is strongly associated with a family history of breast cancer [6]. A study conducted in Jordan about knowledge and practice of breast cancer screening methods also showed that screening uptake was influenced by family history of breast cancer. The participants who had a family history practiced breast cancer screening methods more than participants with no family history [16]. Previous exposure to radiation therapy was significantly associated with the correct age of receiving a mammogram. Individuals who have not received radiation therapy have a risk of lower uptake of mammography. Therefore, it can be conceived that women who received radiation therapy are more likely to be aware of the increased risk of breast cancer, have more accurate knowledge, and follow through with mammogram screening [23].

Limitations

This is a cross-sectional study and the results of this study cannot be generalized because it includes women approaching one healthcare facility. Data that had been collected are from women who accepted to be part of the study, without random selection of participants. Recall bias cannot be excluded because some questions depend on memory. Missing information was there for some of the questions. A language barrier was also encountered.

Recommendations

In light of this, we encourage further national studies to be conducted, to show the prevalence of screening, involving a larger sample size representing the women of UAE. Healthcare providers must encourage and support women to do breast cancer screening when they approach a healthcare facility to educate them about the importance of breast cancer screening. Healthcare professionals play a major role as a primary source of information and were pointed out as a major shortcoming leading to reduced screening uptake in our study. Healthcare facilities can additionally utilize social media forums to distribute awareness to women about breast cancer screening.

## Conclusions

There is a need to increase knowledge and awareness regarding breast cancer screening and mammogram among women in UAE. Breast self-examination should be encouraged and recommended, especially regarding the timing and frequency of screening.

## Figures and Tables

**Figure 4:**
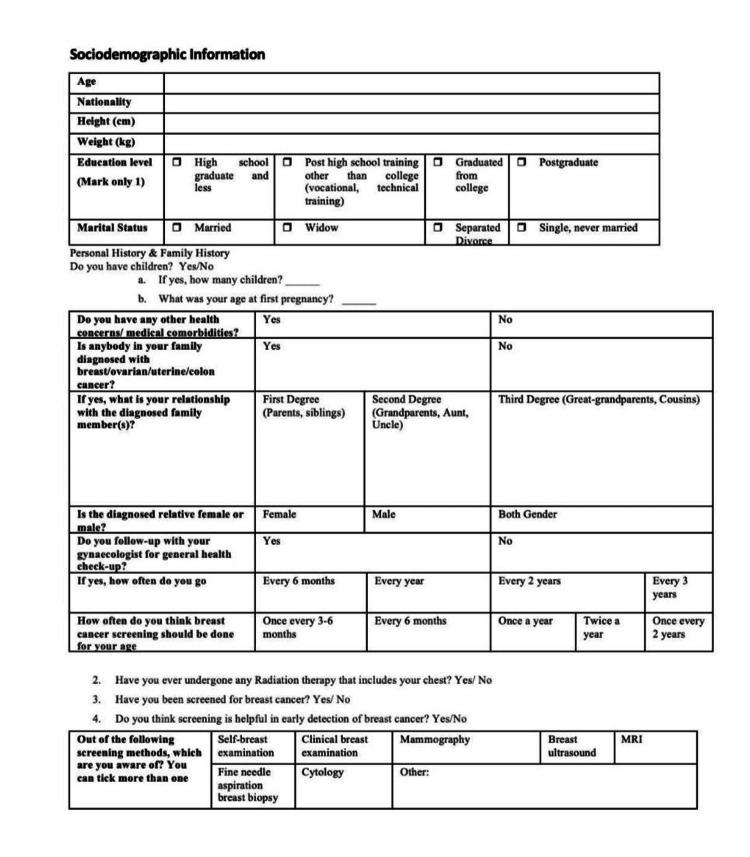
Questionnaire part-A

**Figure 5:**
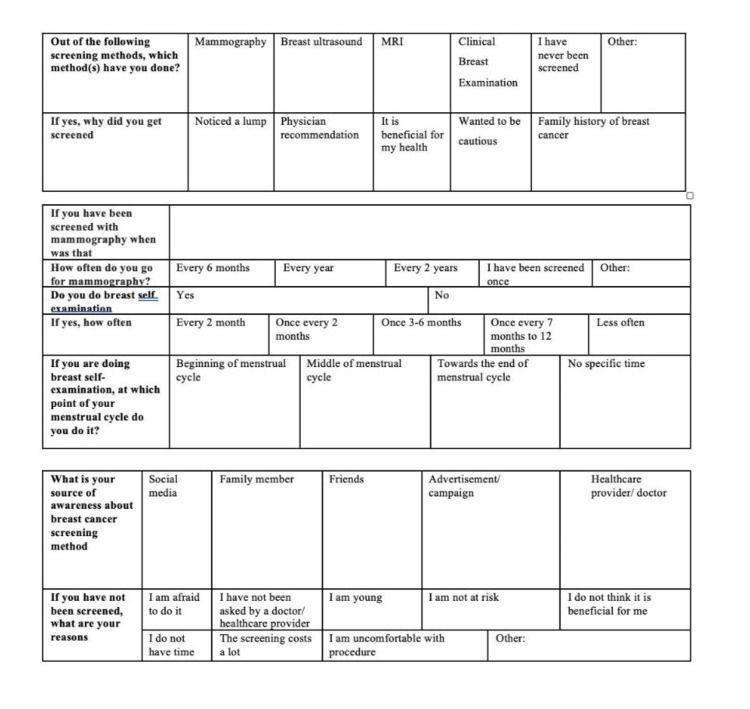
Questionnaire part-B

## Appendices

Appendix material-1
